# Side Effects Associated With Homologous and Heterologous COVID-19 Vaccines: A Cross-Sectional Study in Saudi Arabia

**DOI:** 10.7759/cureus.34030

**Published:** 2023-01-21

**Authors:** Rehab A Mohammed, Rowaid S Yazbik, Lujain H Baajajah, Saud F Alharthy, Hassan Alsalahi, Mohammad A Mahjaa, Mahmoud M Barakat, Mayar I Badawy, Intessar Sultan

**Affiliations:** 1 Department of Internal Medicine, Ibn Sina National College for Medical Studies, Jeddah, SAU; 2 Department of Internal Medicine, Faculty of Medicine for Girls, Al-Azhar University, Cairo, EGY; 3 Department of Medicine, Ibn Sina National College for Medical Studies, Jeddah, SAU; 4 Department of Medicine, Faculty of Medicine for Boys, Al-Azhar University, Cairo, EGY; 5 Department of Clinical Pharmacy, Faculty of Pharmacy, Tanta University, Tanta, EGY

**Keywords:** astrazeneca covid-19 vaccine, homologous and heterologous, side effects of vaccines, covid-19, pfizer-biontech vaccine

## Abstract

Background: Numerous studies on coronavirus disease 2019 (COVID-19) vaccination safety have been conducted in Saudi Arabia. Even though there is less evidence comparing the side effects of different vaccines and a few of them studied the side effects of mixing different platforms of vaccines.

Objectives: This study aimed to evaluate the type and severity of adverse effects following COVID-19 vaccination based on the type and platform of received vaccine and to determine factors that contribute to the occurrence of these side effects.

Methods: This cross-sectional comparative study was conducted in Saudi Arabia from January to the end of February 2022 among COVID-19 vaccine recipients through an online survey. Based on the type of vaccines received, we categorized our participants into two groups - those who received two doses of either the Pfizer or the AstraZeneca COVID-19 vaccines, and those who received mixed vaccination regimen (one dose of Pfizer and one dose of AstraZeneca).

Results: The study included 1,340 participants, of which 56.3% received two doses of the Pfizer vaccine while (7%) received two doses of the AstraZeneca vaccine, and 8.8% received mixed vaccines (one dose of the Pfizer vaccine and one dose of the AstraZeneca vaccine). Pain at the injection site was the most frequent local symptom (37.9%) followed by swelling±redness (27.6%). The local adverse reactions were nearly equal in AstraZeneca and Pfizer vaccines, whereas these were significantly lower in those who received mixed doses (p<0.001). Fever was significantly higher in mixed vaccination regimens compared to other types (p<0.001). The male gender who received the Pfizer vaccine were at higher risk of developing an adverse reaction following vaccination. Unusual side effects (sleep disorders, menstrual irregularities, and symptoms suggestive of diabetic neuropathy) were also reported.

Conclusion: The results suggest the overall safety of Pfizer and AstraZeneca vaccines as well as the mixed vaccination protocol. A heterologous regimen was associated with fewer side effects compared to homologous vaccines. Further studies are needed to assess the long-term side effects.

## Introduction

The emergence of the novel SARS-CoV-2 pandemic mandated the rapid development of vaccines to fight against this pandemic and to save millions of lives [1]. Indeed, different vaccine platforms have been used, some of them are traditional approaches, such as inactivated or live attenuated viruses. Other approaches employ newer platforms, such as recombinant proteins and vectors [2].

These vaccines represented a significant turning point in vaccine production history due to the short development time and the novelty of the used technology as there were no messenger RNA (mRNA) vaccines approved for use in humans prior to BioNTech and Moderna's vaccines [3]. The successful application of mRNA in the coronavirus disease 2019 (COVID-19) pandemic holds promise as a type of replacement therapy to treat a wide range of incurable diseases by introducing mRNA as a vaccine or therapeutic agent [4].

Several reports of COVID-19 vaccines’ side effects have been published worldwide as well as in Saudi Arabia. These reports were variable depending on the type of vaccine investigated and population characteristics, most of them reported relatively common mild-to-moderate side effects [5]. However, a few studies compared the side effects of COVID-19 vaccines, along with the impact of demographic variables, and the presence of comorbidities. Additionally, many of them did not categorize those side effects according to their type (e.g., localized and systemic) and few of them studied the side effects of mixing different types of vaccines [6,7].

Although the COVID-19 pandemic has entered its third consecutive year, the detection of highly transmissible variants raises concern about the consequences of vaccine escape mutations. This condition creates a global demand to intensify vaccination, including booster shots [8].

WHO has recommended the third and fourth COVID-19 booster doses for all people who completed their essential primary doses. Thus, real-world patient-reported data on the adverse effects of COVID-19 vaccines and who is more likely to experience them in our community is still required to support people in making the critical decision to be vaccinated if a booster dose will be required [9].

Therefore, the objective of this study was to evaluate the side effects of Pfizer and AstraZeneca as well as the side effects associated with mixing both vaccines among citizens and residents of Saudi Arabia and to determine factors that contribute to the occurrence of these side effects.

## Materials and methods

Study design, setting, and ethical consideration

A cross-sectional study was conducted from January to the end of February 2022 among the residents of Saudi Arabia. The protocol of the study was submitted to the Ibn Sina National College (ISNC) Research Center Ethics Committee and approval (#REC42/1/139) was obtained before starting the study.

Sampling, study participants, and sample size

According to Epi-info 7.2.5.0 for Windows (Atlanta, GA: CDC), a sample size of approximately 400 was determined based on the following input criteria: margin of error equal to 5%, the design effect is 1, the cluster is 1, and the expected frequency is 50% [10]. The sampling method is a convenient non-probability sampling in which we asked people who are most accessible like family, friends, colleagues, and neighbors in the community. The study included Saudi Arabia resident participants who received a minimum of one dose of either Pfizer or AstraZeneca vaccines. A total of 1,340 responses were received, and all of them completed the survey.

The survey tool

The collection of data was based on an online Google Form questionnaire. The questionnaire is constructed based on a modified version of the questions asked by the Saudi FDA [11] and Centers for Disease Control and Prevention (CDC) official websites which are used for reporting COVID-19-related side effects [12].

The questionnaire was prepared both in English and Arabic languages and revised by medical professionals. The Arabic translation was done by language experts and back-translated by two native speakers to understand any discrepancies. The questionnaire was pilot tested among 50 participants to understand the feasibility and was refined based on feedback. The survey was designed to identify the side effects after receiving a COVID-19 vaccination either local, systemic, allergic, or any uncommon side effects that were related to the vaccines.

The questionnaire included socio-demographic characteristics of the participant and questions about data related to vaccines (type of vaccine received, number of doses, presence, and severity of side effects {using a scale of 1-10}, the time after which they experienced the side effects, need for medical assistance for the treatment of those side effects, time taken to completely recover, and if there is a relationship with medical and non-medical factors) were asked. Health status in terms of chronic illnesses, medications, pregnancy or lactation, or any other vaccines received up to one month before the COVID-19 vaccine was asked. It also included a question on the previous history of allergies and associated history of immunodeficiency or taking medication like high-dose corticosteroids, immunosuppressants, or cancer medicines. Additionally, we asked the participant who has a chronic illness to characterize disease pattern before and after vaccination and indicates any observable changes in terms of symptoms or management. The questionnaire also included an additional section to allow the participants to report other unlisted or uncommon side effects that they may have experienced.

Statistical analysis

The data were analyzed using the IBM SPSS Statistics, released in 2013, version 22.0 for Windows (Armonk, NY: IBM Corp.). Categorical variables were represented as percentages and frequencies, while numerical variables were summarized by calculating the median and interquartile range due to their abnormal distribution. Chi-square and non-parametric Mann-Whitney U tests were used for comparison between different groups. Multivariate linear regression was used to determine the significant predictor of the side effects of vaccines. All results were considered statistically significant at p<0.05.

## Results

Demographic data

The total number of participants studied was 1340. Their median (interquartile range {IQR}) age was 40 (28) years. Around 64% of the participants were females. Most of them were from Saudi (76.2%). A total of 578 (44%) participants reported having at least one non-communicable disease. Obesity was the most comorbidity followed by diabetes and hypertension (14%, 11.5%, and 10.5%, respectively). Seventeen (1.8%) participants were lactating while 10 (1.1%) were pregnant. History of autoimmune diseases or using immunosuppressive drugs was recorded in 16 (1.2%) of the respondents. Nearly 9% of the participants had allergies while 4.7% disclosed having anemia. Most of the participants 967 (72.2%) received two doses of the COVID-19 vaccine, and 373 (27.8%) received only one dose (Table 1).

**Table 1:** Demographic, clinical characteristics, and vaccine data.

Variables		Count (n=1,340)	%
Gender	Female	865	64.6%
	Male	475	35.4%
Occupation	Students/jobless	662	49.4%
	Worker	241	18.0%
	Health care worker	437	32.6%
Nationality	Non-Saudi	319	23.8%
	Saudi	1,021	76.2%
Comorbidities	No	754	56.0%
	Obesity	188	14.0%
	Hypertension	141	10.5%
	Diabetes mellitus	155	11.5%
	Cardiac disease	40	3.0%
	Chest disease	74	5.5%
	Autoimmune/immunosuppression	16	1.2%
	Anemia	63	4.7%
	Allergy	126	9.4%
Pregnancy and lactation at time of vaccination	Pregnant	10	1.1%
	Lactating	17	1.8%
Number of doses	1.0	373	27.8%
	2.0	967	72.2%
1 dose Pfizer-BioNTech C		193	14.4%
1 dose Oxford-AstraZeneca		180	13.4%
2 doses Pfizer-BioNTech C		755	56.3%
2 doses Oxford-AstraZeneca		94	7.0%
Mixed doses		119	8.8%
Age in years, median: 40 (IQR: 28)			

The participants were divided according to the received vaccine into the following two main groups: homologous vaccine recipients (two doses of either Pfizer-BioNTech or AstraZeneca vaccine) and mixed regimen group recipients (one dose Pfizer-BioNTech and one dose AstraZeneca). More than half of the respondents 755 (56.3%) received two doses of the Pfizer-BioNTech vaccine, 94 (7%) received two doses of AstraZeneca while 119 (8.8%) received mixed (AstraZeneca and Pfizer) vaccines (Table 1).

Local reactions

A total of 437 (32.6%) participants reported no local side effects with any vaccine type, while (67.4%) reported at least one local adverse reaction related to the injection site. Pain at the injection site was the most frequent local symptom among vaccine recipients (37.9%) followed by swelling±redness (27.6%). Axillary lymphadenopathy was the least reported side effect (1.9%) of the participants. Most of the local side effects were mild-to-moderate, although 13.8% of the participants declared having severe pain. All reported local reactions were resolved within three days either spontaneously or with analgesics and none of the participants needed any specialized care (Table 2).

**Table 2:** Frequency of local and systemic side effects of the COVID-19 vaccines. COVID-19: coronavirus disease 2019; NSAIDs: non-steroidal anti-inflammatory drugs

Variables		Count (n=1,340)	%
Local symptoms		-	-
No local symptoms		437	32.6%
Pain		487	32.9%
Pain-swelling±redness		357	27.6%
Local lymphadenopathy±other local symptoms		25	1.9%
Onset of pain	Within the first 15 min	103	7.7%
	From 1 to 24 h	578	43.1%
	Between 24 and 48 h	203	15.1%
	After 48 h	14	1.0%
The dose with more pain	After 1st dose	176	18.2%
	After 2nd dose	184	19.0%
	Both equal	265	27.4%
Severity of symptoms	Mild	273	30.4%
	Moderate	501	55.8%
	Severe	124	13.8%
Treatment	Paracetamol	478	35.6%
	NSAIDs	30	2.2%
	Paracetamol, NSAIDs	32	2.3%
	Local treatment (cold compressors-local analgesics)	4	0.3%
	No treatment	803	59.6%
Systematic symptom		-	-
No systemic symptoms		455	33.8%
Fever		513	38.1%
Fatigue		186	13.8%
Pains (body pain, muscle pains, headache)		142	10.5%
Rigors, palpitations, dyspnea		41	3.0%
Others (nausea, vomiting, cramp)		10	0.7%
The dose with most systemic symptoms	1st dose	176	18.2%
	2nd dose	185	19.1%
	Both	266	27.5%
Onset	Less than 15 min	1	0.1%
	From 1 to 24 h	542	40.4%
	Between 24 and 48 h	149	11.1%
	After 48 h	10	0.7%
Severity of symptoms	Mild	275	20.5%
	Moderate	501	37.3%
	Severe	124	9.2%
Duration in days, median: 3 (IQR: 2)			

The local adverse reactions were nearly equal in both vaccines, whereas local effects were less likely to appear with mixed doses (p<0.001). Lymphadenopathy was reported in both vaccines with a slightly higher prevalence in the Pfizer vaccine, on the other hand, no recorded lymph node enlargement in those who received mixed vaccines (Table 3).

**Table 3:** Local and systemic reactions related to type of vaccine.

Variables	Pfizer	AstraZeneca	Mixed (119)	p-Value
Local reactions	-	-	-	<0.001
No reactions	297 (31.3%)	84 (30.7%)	56 (47%)	
Pain and tenderness	609 (64.0%)	179 (65%)	58 (48.7%)	
Redness and swelling	23 (2.4%)	6 (2.2%)	5 (4.2%)	
Lymph nodes	21 (2.2%)	5 (1.8%)	0	
Systemic symptoms	-	-	-	
No symptoms	361 (38.0%)	57 (20.8%)	30 (25.4%)	
Rigor, palpitations, and dyspnea	32 (3.4%)	-	-	
Pains and headache	119 (12.5%)	13 (4.7%)	10 (8.5%)	
Fever	290 (30.6%)	151 (55.1%)	72 (61%)	
Fatigue	141 (14.9)	42 (15.3%)	-	
Nausea, diarrhea	-	3 (1.1%)	1 (0.1%)	

Systemic reactions

Fever was the most common systemic reaction followed by fatigue and pain (38.1%, 13.8%, and 10.5%, respectively). The last reported systemic side effects were nausea (10.2%) and diarrhea (6.7%) (Table 2). Fever was more prevalent in those who received mixed doses (61%) followed by Oxford/AstraZeneca (55.1%) while a lower percentage of Pfizer-BioNTech recipients developed fever (30.6%) (p<0.001). Rigors, palpitations, and dyspnea were only reported with the Pfizer vaccine (3.4%). Other systemic manifestations including headache, joints, and muscle pains were higher in Pfizer-BioNTech recipients compared to other Oxford-AstraZeneca and mixed vaccines (12.5%, 4.7%, 8.5%, respectively) (p<0.001) (Table 3).

Allergic reactions after vaccination

There was a history of allergy in 126 participants (9.4%) (Table 1). However allergic reactions after vaccination were experienced by only 45 (3.35%) participants with higher frequency in the second dose compared with the first dose. Twenty-one (1.6%) participants had to visit the doctor after the onset of symptoms. Severe allergic manifestations that needed hospitalization were necessary in one case after the first Pfizer-BioNTech dose and two cases following Oxford-AstraZeneca, one after the first and another one after the second dose (Table 4).

**Table 4:** Allergic reactions after vaccination. KCO: known case of allergy

Variables		Total count=1340, KCO=126	%
Skin rash-urticaria		18 (8)	1.3%
Chest symptoms		15 (6)	1.0%
Swollen tongue, lips, or face		3 (0)	0.2%
Both skin rash/urticaria, chestanti		5 (2)	0.4%
Skin, chest, swollen tongue, lips, or face		3 (0)	0.1%
Total allergy		45 (16)	3.4%
The dose with more severe allergy	1st dose	16	1.2%
	2nd dose	22	1.6%
	Both	7	0.5%
Onset	<15 min	7	0.5%
	<24 h	20	1.5%
	24-48 h	13	1%
	After 48 h	10	0.7%
Severity	Mild	12	0.9%
	Moderate	25	1.9%
	Severe	13	1.0%
Treatment	Consultation and prescription	21	1.6%
	Over-the-counter anti-histaminic	4	0.3%
	Treatment at hospital	3	0.2%
Duration in days, median: 1 (IQR: 1)			

Other reported side effects

Surprisingly, 221 participants out of 755 reported disturbed sleep rhythm and nightmares after the first doses of the Pfizer vaccine that did not alleviate until the end of the study. Out of 155 diabetic individuals, 16 diabetic patients suffered from increased blood sugar levels that required higher doses of the anti-diabetic drugs and nine of them reported neuropathic-like manifestations after vaccination. Menstrual irregularities that extended up to four months were observed by 305 women (Table 5).

**Table 5:** Other reported side effects.

Disturbed sleep rhythm and nightmares	221 out of 755	29.3%
Menstrual irregularities	305 (out of 865)	35.2%
Impaired glucose level in diabetics	16 (out of 155)	10.23%
Neuropathy in diabetics	9 (out of 155)	5.8%

Risk factors for vaccines side effect

A logistic regression analysis was performed to evaluate factors that can potentially be associated with adverse effects after COVID-19 vaccination. The model was based on multiple variables including age, gender, occupation, nationality, presence of chronic diseases, known allergy, pregnancy, or lactation at the time of vaccination, and the type of vaccine. The model was statistically significant, χ^2^ (12)=33.443, p<0.001. The model explained 4.8% (Nagelkerke R^2^) of the variances’ side effects and correctly classified 56.4% of cases. Significant predictors of vaccines’ side effects were gender (p<0.01), occupation (p<0.05), and type of vaccination (p<0.05). Male workers who received the Pfizer vaccine showed a significantly higher frequency of side effects (Table 6).

**Table 6:** Determinants of side effects of vaccines. EXP: exponential function

Variables	p-Value	OR	95% CI for EXP (B)	
			Lower	Upper
Pfizer	0.039	-	-	-
Oxford	0.607	1.124	.721	1.753
Mixed	0.037	1.720	1.034	2.860
Age	0.170	1.244	.911	1.699
Gender	0.004	2.096	1.272	3.453
Jobless	0.013	-	-	-
Worker	0.004	0.623	0.454	0.856
Health care worker	0.071	0.687	0.456	1.033
Nationality	0.156	0.787	0.565	1.096
Chronic diseases	0.863	1.049	0.608	1.808
Pregnancy	0.219	3.206	0.500	20.566
Known allergy	0.285	0.792	0.516	1.215

## Discussion

The Pfizer and AstraZeneca vaccines are authorized as homologous 2 doses regimens, however, heterologous combinations of vector and mRNA vaccines are already been implemented in many countries including Saudi Arabia [13]. In this study, 119 (8.8%) of the participants received mixed regimens (one dose of the Pfizer vaccine and another dose of the AstraZeneca vaccine). Mixing vaccines of different platforms is associated with more stimulation of cellular immune response and higher neutralizing antibodies so this strategy may improve the vaccines' effectiveness in addition to solving the problem of vaccine shortage in many regions [13]. However, this vaccination strategy is not well understood in terms of efficacy and safety. Therefore, in this study, we aimed to compare the type and frequency of adverse events following the administration of two doses of homologous vaccine with those who received the mixed vaccination approach among Saudi residents and to assess factors that contribute to their occurrence.

The present study showed that around 66% of the studied participants experienced either local or systemic adverse reactions after COVID-19 vaccination. Pain followed by redness and swelling at the injection site was the most frequent local symptom among vaccine recipients which is nearly equal after the first and the second doses of the vaccines and both types of vaccines.

Vaccines, irrespective of their composition, induce some inflammation at the injection site within the first hours, contributing to pain, redness, and swelling. It is a common concept that pain at the site of injection is a predictive sign of a good vaccine response, however, limited data either support or contradict this concept [14].

In our survey, about 18% of the participants described their local symptoms as severe with 2.8% of them requiring medical consultation but none of them needed hospital admission. Overpenetration, wrong site, and wrong technique may be implicated as possible mechanisms of injection pain [15].

Lymphadenopathy was reported during the clinical trials following the COVID-19 vaccination. In the Pfizer vaccine safety trials, only 0.3% of the recipients reported lymphadenopathy [16], while the AstraZeneca vaccine phase 3 clinical trials did not report any cases of lymphadenopathy [17]. In our study, local lymphadenopathy after COVID-19 vaccines were reported by nearly 2% of the participants who received either the Pfizer or AstraZeneca vaccine with a slightly higher rate in the Pfizer vaccine while no lymph node enlargement was reported in those who received mixed vaccines.

Cohen et al. reported lymphadenopathy in 45.6% of the participants [18]. while McMurry et al. reported a significantly lower rate of lymphadenopathy in the vaccinated group compared to the non-vaccinated control group [19]. These contradictory results may be due to the selection process of the participants and the nature of the study.

Concerning the type of vaccine, fever was more common in those who received mixed types of vaccines followed by the AstraZeneca vaccine while rigors, palpitation, and dyspnea were only reported in the Pfizer vaccine recipients. Our findings are in line with the Com-COV study which found that fever was more prevalent in those who received the mixed vaccines compared with participants who received two doses of the same vaccine (34% and 10%, respectively) [20]. Another study in Saudi Arabia found that a mixed vaccination approach was associated with more side effects than the matched vaccination approach [9]. Other systemic manifestations including nausea and vomiting were only experienced in AstraZeneca vaccine recipients.

Immunologically mediated allergic reactions can cause various manifestations ranging from skin disorders to life-threatening systemic reactions [21]. In this study, only 45 individuals (3.5%) reported allergic manifestations, but three persons reported severe allergic reactions that needed hospital admission. Allergic reactions to vaccines are commonly due to the components of vaccines or attached elements like egg protein and gelatin. The Pfizer-BioNTech vaccine included two new lipid nanoparticles, one is polyethylene glycol with a molecular weight of 2,000 Da (PEG2000) which is reported to cause allergic reactions in some studies [22]. The international consensus didn't recommend routine skin tests with COVID-19 vaccines for the purpose of vaccine withholding, they claimed that the sensitivity of skin tests in predicting serious hypersensitivity reactions to COVID-19 vaccines is unclear [23]. While past histories of allergies were noted to have a higher risk of allergies to vaccines, in our study, a history of allergies was not a risk factor for allergic reactions post-COVID-19 vaccination [24].

Uncommon side effects were also reported in this study, 16 diabetic patients suffered from increased blood sugar levels that required higher doses of the anti-diabetic drugs and nine of them described symptoms constant with neuropathy after vaccination. Safavi et al. identified 23 patients with new-onset neuropathic symptoms following COVID vaccination, one of them was vaccinated by AstraZeneca and 12 received the Pfizer vaccine [25].

Most of the cross-sectional studies reported menstrual abnormalities in a significant number of women up to 50% after the COVID-19 vaccine. Nonetheless, they were unable to report the causal relationship between menstrual irregularities and COVID-19 vaccination [26]. In this study, menstrual irregularities were identified in 305 women (35.2%), these irregularities extended up to four months after vaccination.

In contrast to our study, a retrospective survey in Saudi Arabia reported a low incidence of menstrual abnormalities following COVID-19 vaccination, they hypothesized that platelet disorders or possible hormonal disturbances could be the cause for these abnormalities [27]. Sleep disorder was one of the most frequently reported adverse events in our study, with up to 29.3% of cases complaining of sleepiness following COVID-19 vaccination. Garrido-Suárez et al. proposed that COVID-19 vaccines may stimulate the release of pro-inflammatory cytokines that activate GABAergic neurons providing an inhibitory effect on orexinergic neurons [28]. These reported uncommon side effects should be considered in pharmacovigilance future research to understand the potential mechanisms of these side effects and to preserve confidence in these novel vaccines.

The strength of this study is the comparison of the adverse effects between homologous COVID-19 vaccine regimens with heterologous combinations. The main limitations of this study are the self-reported adverse events and a cross-sectional design that doesn’t allow for causality interpretation. Another limitation is the lack of information regarding the history of SARS-CoV-2 infection, which could be another risk factor for developing adverse effects. Further cohort studies on a large scale are recommended to compare the side effects of a group receiving matched doses and a group receiving mixed doses of COVID-19 vaccines. Additionally, the study evaluated short-term adverse events. Therefore, further cohort studies are recommended to investigate the long-term adverse events following the administration of these COVID-19 vaccines.

## Conclusions

Our findings indicate that heterologous and homologous COVID-19 vaccination regimens are associated with different patterns and number of side effects. Both regimens have acceptable safety. A heterologous regimen was associated with fewer side effects compared to homologous vaccines. These results could be helpful in designing or modifying future vaccination plans. Additional studies on the efficacy and immune responses induced by heterologous vaccines are needed.

## References

[1] Khan WH, Hashmi Z, Goel A (2021). COVID-19 pandemic and vaccines update on challenges and resolutions. Front Cell Infect Microbiol.

[2] Acosta-Coley I, Cervantes-Ceballos L, Tejeda-Benítez L (2022). Vaccines platforms and COVID-19: what you need to know. Trop Dis Travel Med Vaccines.

[3] Verdecia M, Kokai-Kun JF, Kibbey M, Acharya S, Venema J, Atouf F (2021). COVID-19 vaccine platforms: Delivering on a promise?. Hum Vaccin Immunother.

[4] Qin S, Tang X, Chen Y (2022). mRNA-based therapeutics: powerful and versatile tools to combat diseases. Signal Transduct Target Ther.

[5] Menni C, Klaser K, May A (2021). Vaccine side-effects and SARS-CoV-2 infection after vaccination in users of the COVID symptom study app in the UK: a prospective observational study. Lancet Infect Dis.

[6] Alhowaymel F, Abdelmalik MA, Mohammed AM, Mohamaed MO, Alenezi A (2022). Reported side effects of COVID-19 vaccination among adults in Saudi Arabia: a cross-sectional study. SAGE Open Nurs.

[7] Almughais ES, Alharbi AH, Aldarwish HA, Alshammari AF, Alsuhaymi RS, Almuaili JA, Alanizy AM (2022). Side-effects of COVID-19 vaccines among the Saudi population: a cross-sectional study. Saudi Med J.

[8] Dolgin E (2021). Omicron is supercharging the COVID vaccine booster debate. Nature.

[9] (2022). Statement for healthcare professionals: how COVID-19 vaccines are regulated for safety and effectiveness. https://www.who.int/news/item/17-05-2022-statement-for-healthcare-professionals-how-covid-19-vaccines-are-regulated-for-safety-and-effectiveness.

[10] Carstensen B, Plummer M, Laara E, Hills M (2022 (2022). Epi: statistical analysis in epidemiology. https://cran.r-project.org/web/packages/Epi/index.html.

[11] Side effects of corona vaccines (COVID-19). https://ade.sfda.gov.sa/Covid/CovidRequest.

[12] Selected adverse events reported after COVID-19 vaccination. https://www.cdc.gov/coronavirus/2019-ncov/vaccines/safety/adverse-events.html.

[13] Alshahrani MM, Alqahtani A (2022). Side effects of mixing vaccines against COVID-19 infection among Saudi Population. Vaccines (Basel).

[14] Mitchell TC, Casella CR (2017). No pain no gain? Adjuvant effects of alum and monophosphoryl lipid A in pertussis and HPV vaccines. Curr Opin Immunol.

[15] Hesse EM, Atanasoff S, Hibbs BF (2020). Shoulder injury related to vaccine administration (SIRVA): petitioner claims to the National Vaccine Injury Compensation Program, 2010-2016. Vaccine.

[16] Polack FP, Thomas SJ, Kitchin N (2020). Safety and efficacy of the BNT162b2 mRNA COVID-19 vaccine. N Engl J Med.

[17] Voysey M, Clemens SA, Madhi SA (2021). Safety and efficacy of the ChAdOx1 nCoV-19 vaccine (AZD1222) against SARS-CoV-2: an interim analysis of four randomised controlled trials in Brazil, South Africa, and the UK. Lancet.

[18] Cohen D, Krauthammer SH, Wolf I, Even-Sapir E (2021). Hypermetabolic lymphadenopathy following administration of BNT162b2 mRNA Covid-19 vaccine: incidence assessed by [18F]FDG PET-CT and relevance to study interpretation. Eur J Nucl Med Mol Imaging.

[19] McMurry R, Lenehan P, Awasthi S (2021). Real-time analysis of a mass vaccination effort confirms the safety of FDA-authorized mRNA COVID-19 vaccines. Med (N Y).

[20] Shaw RH, Stuart A, Greenland M, Liu X, Nguyen Van-Tam JS, Snape MD (2021). Heterologous prime-boost COVID-19 vaccination: initial reactogenicity data. Lancet.

[21] (2021). Allergic reactions including anaphylaxis after receipt of the first dose of Moderna COVID-19 vaccine - United States, December 21, 2020-January 10, 2021. MMWR Morb Mortal Wkly Rep.

[22] Wenande E, Garvey LH (2016). Immediate-type hypersensitivity to polyethylene glycols: a review. Clin Exp Allergy.

[23] Greenhawt M, Abrams EM, Shaker M (2021). The risk of allergic reaction to SARS-CoV-2 vaccines and recommended evaluation and management: a systematic review, meta-analysis, GRADE assessment, and international consensus approach. J Allergy Clin Immunol Pract.

[24] Bian S, Li L, Wang Z, Cui L, Xu Y, Guan K, Zhao B (2022). Allergic reactions after the administration of COVID-19 vaccines. Front Public Health.

[25] Safavi F, Gustafson L, Walitt B (2022). Neuropathic symptoms with SARS-CoV-2 vaccination. medRxiv.

[26] Nazir M, Asghar S, Rathore MA (2022). Menstrual abnormalities after COVID-19 vaccines: a systematic review. Vacunas.

[27] Alghamdi AN, Alotaibi MI, Alqahtani AS, Al Aboud D, Abdel-Moneim AS (2021). BNT162b2 and ChAdOx1 SARS-CoV-2 post-vaccination side-effects among Saudi vaccinees. Front Med (Lausanne).

[28] Garrido-Suárez BB, Garrido-Valdes M, Garrido G (2022). Reactogenic sleepiness after COVID-19 vaccination. A hypothesis involving orexinergic system linked to inflammatory signals. Sleep Med.

